# Influence of *Tilia tomentosa* Moench Extract on Mouse Small Intestine Neuromuscular Contractility

**DOI:** 10.3390/nu13103505

**Published:** 2021-10-04

**Authors:** Silvia Cerantola, Sofia Faggin, Gabriela Annaloro, Federica Mainente, Raffaella Filippini, Edoardo Vincenzo Savarino, Anna Piovan, Gianni Zoccatelli, Maria Cecilia Giron

**Affiliations:** 1 Department of Pharmaceutical and Pharmacological Sciences, University of Padova, 35131 Padova, Italy; silvia.cerantola@unipd.it (S.C.); sofia.faggin@phd.unipd.it (S.F.); gabry.annaloro@gmail.com (G.A.); raffaella.filippini@unipd.it (R.F.); anna.piovan@unipd.it (A.P.); 2 Department of Biotechnology, University of Verona, 37134 Verona, Italy; federica.mainente@univr.it (F.M.); gianni.zoccatelli@univr.it (G.Z.); 3 Department of Surgery, Oncological and Gastrointestinal Science, University of Padova, 35121 Padova, Italy; edoardo.savarino@unipd.it; 4 IRCCS San Camillo Hospital, 30126 Venice, Italy

**Keywords:** functional gastrointestinal disorders, linden flower, enteric nervous system, intestinal contractile response, *Tilia tomentosa* Moench, cholinergic neurotransmission, nitrergic pathways

## Abstract

Functional gastrointestinal disorders (FGIDs) are characterized by abdominal pain, bloating and bowel disturbances. FGID therapy is primarily symptomatic, including treatment with herbal remedies. Flower extract of *Tilia tomentosa* Moench (*TtM*) is occasionally used as an anti-spasmodic in popular medicine. Since its effect on intestinal response is unknown, we evaluated the influence of *TtM* extract on small intestine contractility. Ileal preparations from C57BL/6J mice were mounted in organ baths to assess changes in muscle tension, following addition of *TtM* extract (0.5–36 μg/mL) or a vehicle (ethanol). Changes in contractile response to receptor- and non-receptor-mediated stimuli were assessed in ileal preparations pretreated with 12 μg/mL *TtM*. Alterations in the enteric nervous system neuroglial network were analyzed by confocal immunofluorescence. Increasing addition of *TtM* induced a marked relaxation in ileal specimens compared to the vehicle. Pretreatment with *TtM* affected cholinergic and tachykininergic neuromuscular contractions as well as K^+^-induced smooth muscle depolarization. Following incubation with *TtM*, a significant reduction in non-adrenergic non-cholinergic-mediated relaxation sensitive to Nω-Nitro-L-arginine methyl ester hydrochloride (pan-nitric oxide synthase inhibitor) was found. In vitro incubation of intestinal specimens with *TtM* did not affect the myenteric plexus neuroglial network. Our findings show that *TtM*-induced intestinal relaxation is mediated by nitric oxide pathways, providing a pharmacological basis for the use of *TtM* in FGIDs.

## 1. Introduction

Functional gastrointestinal disorders (FGIDs), such as irritable bowel syndrome (IBS), are characterized by abdominal pain, constipation, bloating, and diarrhea, and often arise following lifestyle disturbances, physical or/and emotional stress, or drug therapy, determining about a third of gastroenterology examinations [1]. One of the factors contributing to the pathogenesis of FGIDs appears to be a dysfunction of the autonomic nervous system however, i.e., the etiology is complex and multi-factorial. Antispasmodic agents and gut-brain neuromodulators are widely used for the treatment of FGIDs with no clear relative efficacy [2].

*Tilia tomentosa* Moench (*TtM*) (synonym: *Tilia argentea* Desf. ex DC.), commonly known as silver linden or silver lime, is a deciduous tree native of southeastern Europe and Asia that belongs to the Malvaceae family [3]. Several *Tilia* species are used in folk medicine, including *Tilia americana* and *Tilia mexicana* [4,5,6]. Liquid preparations derived from *Tilia* spp. buds, young sprouts, flowers, or leaves have been used as mild antispasmodic, diaphoretic, sedative, or anti-inflammatory agents for the treatment of gastrointestinal (GI) disorders, flu, migraine, and anxiety in traditional and complementary medicine [3,7,8,9,10].

In the last decade, several studies have reported the ability of *TtM* to induce sedative effects on the central nervous system, potentially through the interaction of their phenolic content, mainly represented by flavonol glycosides (quercetin, kaempferol, and apigenin derivatives) and phenolic acids, on GABAergic and serotoninergic pathways [6,7,8,11]. Interestingly, the oral consumption of flavonoids has been shown to preserve the gut barrier integrity and GI activity as well as to exert a modulatory role on gut microbiota by regulating toll-like receptor signaling [12,13] highlighting the potential beneficial properties of these compounds in FGIDs. 

To date, investigations on *TtM*-mediated effects on intestinal contractility are missing, although *TtM* is widely used in gemmotherapy and is recognized to be rich in flavonoids. Therefore, in this study we aimed to determine whether *TtM* influences contractile smooth muscle function, using an isolated mouse ileum model. Furthermore, we assessed the impact of *TtM* on the enteric nervous system (ENS) structure and glial reactivity to ensure its safety.

## 2. Materials and Methods

### 2.1. Drugs and Solutions

Krebs solution (118 mM NaCl, 4.7 mM KCl, 2.5 mM CaCl_2_·2H_2_O, 1.2 mM KH_2_PO_4_, 1.2 mM MgSO_4_·7H_2_O, 25.0 mM NaHCO_3_, and 11 mM glucose), oxygenated with 95% O_2_/5% CO_2_, was used for the organ bath experiments. Unless otherwise specified, all chemicals were obtained from Sigma–Aldrich (Milan, Italy) and were of the highest commercially available analytical grade. Paraformaldehyde (PFA) was purchased from Electron Microscopy Sciences–Società Italiana Chimici (Rome, Italy), and Triton-X-100 was obtained from Applichem (Milan, Italy).

### 2.2. Plant Material and Extract Preparation of Tilia tomentosa Moench 

*TtM* extract was kindly supplied by Agripharma (Padua, Italy). It was obtained following Ph. Eur. indications by extraction of dry linden flowers with a 50% (*v:v*) ethanol solution, followed by vacuum concentration. The final product was characterized by a drug/solvent ratio of 1:1 (*w:v*) and ethanol concentration of 30% (*v:v*). 

### 2.3. Chemical Analysis

Analyses were performed using an Agilent 1100 HPLC Series System (Agilent, Santa Clara, CA, USA) equipped with a degasser, quaternary gradient pump, column thermostat, and UV-Vis detector. A Gemini 5 µm C6-Phenyl column (250 × 4.6 mm) from Phenomenex (Torrance, CA, USA) was employed, at 40°C. The mobile phase consisted of 0.1% acetic acid in water (A) and acetonitrile (B), with the following gradient elution program: 97% A at 0–6 min, 75% A at 15 min, 75% A at 20 min, and 20% A at 30 min. The flow rate was 1 mL min^-1^, with an injection volume of 10 µL; detection was at 280 and 365 nm; spectra were acquired from 200 to 800 nm. The extract hydrolysis was performed according to Innocenti et al. [14]. 

The content of flavonols was expressed as quercetin and kaempferol derivatives. Quercetin and kaempferol standard solutions (1 mg/mL) were prepared in methanol, and the calibration curves were obtained in a concentration range of 5–100 μg/mL, with six concentration levels. Peak areas were plotted against corresponding concentrations (*R^2^*= 0.9998, 0.9996). 

### 2.4. Effects of Tilia tomenstosa Moench on Isolated Mouse Ileum 

All animal care and experimental procedures were approved by the Animal Care and Use Ethics Committee of the University of Padova and by the Italian Ministry of Health (authorization number: 41451.N.NRD, 11 January 2020) and were performed in accordance with national and EU guidelines for the handling and use of experimental animals. In vitro contractile and relaxing ileal responses were measured using the isolated tissue bath technique as previously described [15,16]. Full-thickness 1 cm distal ileum segments were isolated from adult male C57BL/6 J mice (6±1 months old; Charles River Laboratories) and mounted in 10 mL organ baths and allowed to equilibrate for 45 min in Krebs solution, maintained at 37 °C and oxygenated with 95% O_2_/5% CO_2_, in a resting tension of 0.5 g. The mechanical activity of ileum segments was recorded by isometric transducers (World Precision Instruments, Berlin, Germany) connected to a PowerLab 4/30 system (ADInstruments, Oxford, UK). After 30 min equilibration, ileal segments were stretched passively to an initial tension of 0.1 g and brought to their optimal point of length-tension relationship using 1 μM carbachol (CCh). To verify the effects of *TtM* on basal small intestinal tension, the preparations were incubated with *TtM* extract or ethanol vehicle. Increasing concentrations of *TtM* (0.5–36 µg/mL, in terms of flavonol equivalents, i.e., quercetin + kaempferol) or the vehicle were added to the organ bath cumulatively to generate full concentration response curves. Then, to further investigate the effect of *TtM*, ileal tissues, pretreated for 15 min with 12 µg/mL ethanol extract or the vehicle, were exposed to: (i) increasing concentration of CCh (0.001–100 µM) or electrical field stimulation (EFS, 0–40 Hz; 1-ms pulse duration; 10 s pulse trains, 40 V) using platinum electrodes connected to an S88 stimulator (Grass Instrument) to evaluate excitatory cholinergic response; (ii) 60 mM KCl to asses non-receptor-mediated contractile responses; (iii) non-adrenergic non-cholinergic (NANC) conditions, obtained by 20 minute-preincubation with 1 µM atropine (a muscarinic receptor antagonist) + 1 µM guanethidine (an adrenergic neuron antagonist), followed by treatment with 100 µM Nω-Nitro-L-arginine methyl ester hydrochloride (L-NAME, a non-selective nitric oxide synthase (NOS) inhibitor), to reveal the inhibitory nitrergic relaxation; (iv) 10 Hz EFS-mediated contraction in presence of 100 μM L-NAME under NANC conditions, to uncover tachykininergic-mediated contractions. Contractile responses were expressed as gram tension/gram dry tissue weight of ileal segments, and ileal relaxation was calculated as AUC and normalized per gram dry tissue weight and expressed as a percentage [17].

### 2.5. Immunohistochemistry on Ileal Whole-Mount Preparations

To assess potential influences on the architecture of the enteric neuroglial network, fresh isolated distal ileum 10 cm segments were subjected to 1 h incubation with or without 12 µg/mL *TtM*, dissolved in Krebs solution. Ileal segments were then rinsed with phosphate buffered saline (PBS) and exposed to fixative solution (4% PFA in PBS) for 2 h at room temperature. After washes in PBS (3 × 15 min), ileal segments were cut in 0.5 cm-pieces opened along the mesenteric border and placed as a flat sheet to the bottom of Sylgard-coated dishes, with the mucosal side down. Using a dissecting microscope, longitudinal muscle-myenteric plexus whole-mount preparations (LMMPs) were isolated as previously described [15]. 

LMMP preparations were gently pinned down on a Sylgard-coated dish and gently washed with PBT (PBS with 0.3% Triton X-100) for 45 min. After blocking nonspecific-binding sites with 5% bovine serum albumin (BSA) in PBT for 1 h at room temperature, LMMPs were incubated overnight at room temperature with primary antibodies (Table 1) diluted in PBT and 5% BSA. LMMPs were then washed and incubated at room temperature for 2 h with secondary antibodies (Table 1) diluted in PBT and BSA 5%. LMMPs were mounted on glass slides using a Mowiol mounting medium (Citifluor™ Mountant Solution AF1) and stored in the dark at –20 °C until analysis. Immunohistochemistry process and specificity of antibodies, as positive controls on tissue expressing the protein/antigen, were assessed previously [15,17,18,19,20]. Negative controls were obtained by incubating sections with isotype-matched control antibodies at the same concentration as the primary antibody and/or pre-incubating each antibody with the corresponding control peptide (final concentration as indicated by manufacturer’s instructions) [19,21].

### 2.6. Imaging Acquisition and Analysis

Images were acquired with the Zeiss LSM 800 confocal imaging system (Oberkochen, Germany) equipped with an oil-immersion 63× objectives (NA 1.4). Z-series images (25 planes) of 512 × 512 pixels were captured and processed as maximum intensity projections. All microscope settings were set to collect images below saturation and were kept constants for all images. The number of HuC/D^+^ or nNOS^+^ neurons gathered was normalized to the total myenteric ganglia area, as previously described [22,23]. Changes in the immunoreactivity for S100β were determined by evaluating the density index of labeling per myenteric ganglia area and were reported as mean ± SEM [15].

### 2.7. Statistical Analysis

Data were analyzed using GraphPad Prism 8.4 (San Diego, CA, United States) and are expressed as mean ± SEM. The distribution of data was tested with the Shapiro-Wilk normality test. Differences between the experimental groups were assessed using paired or unpaired Student’s t-test and one-way analysis of variance (ANOVA), followed by the post hoc Bonferroni test. The results were considered statistically significant at *p* < 0.05; “N” indicates the number of ileal segments.

## 3. Results

### 3.1. Phytochemical Analysis 

**Figure 1:**
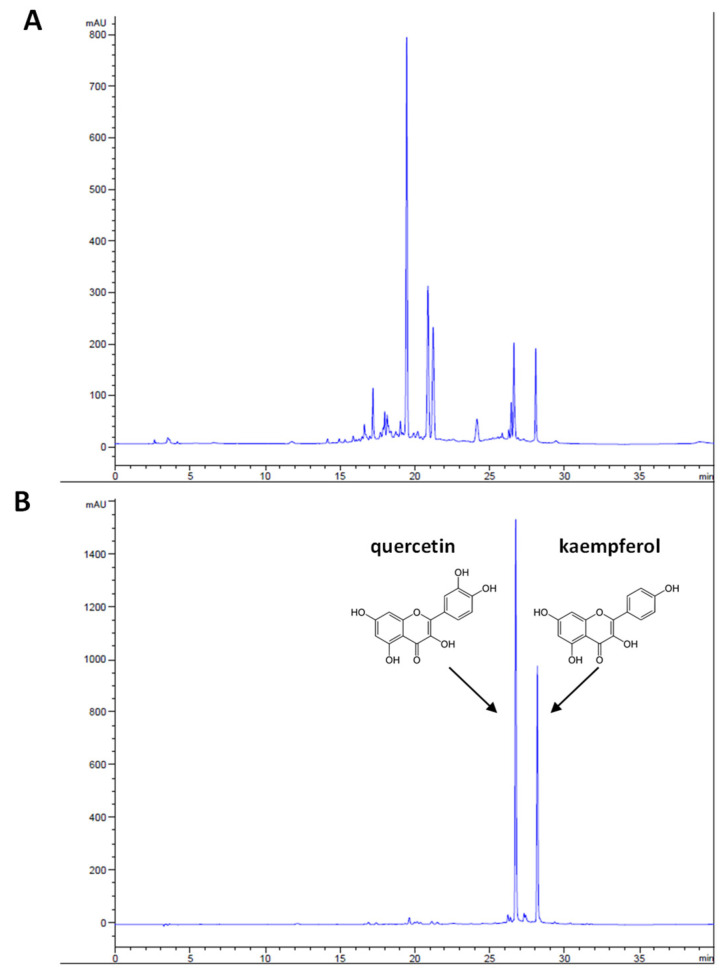
HPLC chromatograms of *Tilia tomentosa* Moench extract, recorded at 365 nm. The upper chromatogram (**A**) shows the extract profile, whereas the lower one (**B**) depicts the hydrolyzed extract profile, identifying the peaks of quercetin and kaempferol at 26.7 and 28.1 min, respectively, as well as their chemical structures.

HPLC-UV Vis analysis allowed us to obtain a chromatographic fingerprint of the extract and to define marker compounds for the quantitative analyses. In the extract, several polyphenols were revealed. The analysis of the hydrolyzed extract allowed us to identify quercetin and kaempferol as the dominant aglycones (Figure 1). Based on these results, the quantitative analyses referred to quercetin and kaempferol derivatives. The contents of quercetin and kaempferol derivatives in the extract were 399 and 252 µg/mL, respectively.

### 3.2. Tilia Tomentosa Inhibits Basal Contractile Responses of Isolated Small Intestine Preparations

**Figure 2:**
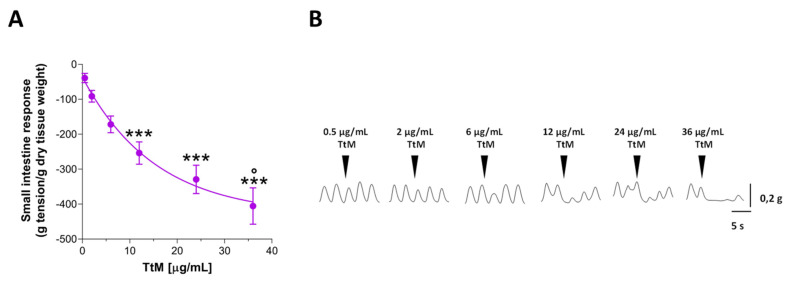
Effect of *Tilia tomentosa* Moench (*TtM)* extract on small intestine contractility. (**A**) Ileal response induced by increasing quantity of *TtM* extract in ileal preparations. (**B**) Representative tracings of responses induced by increasing quantity of *TtM* in ileal preparations. Data are reported as mean ± SEM. *** *p* < 0.001 vs 0.5 µg/mL *TtM*; ° *p* < 0.05 vs. 12 µg/mL *TtM*.

The addition of increasing concentrations of *TtM* (0.5–36 µg/mL) determined the complete disappearance of spontaneous contraction amplitude of isolated ileal segments in a concentration-dependent manner (Figure 2). Since no previous study has been conducted on murine ileal preparations, the concentrations were determined experimentally. The inhibitory effect of *TtM* extract was found to be significant at 12, 24 and 36 µg/mL, determining a 6.6- (*p* < 0.001), 8.6- (*p* < 0.001), and 10-fold (*p* < 0.001) increase of inhibition of ileum contraction, respectively (Figure 2). However, there was no significant difference between the effect mediated by *TtM* at 12 µg/mL and 24 µg/mL. Therefore, the concentration of the extract at 12 µg/mL was used in the subsequent experiments. Interestingly, following the treatment with *TtM*, the relaxed ileal tissues immediately recovered after washing with Krebs solution, to reveal that the relaxant effect is reversible and not a consequence of irreversible tissue damage. 

### 3.3. Tilia Tomentosa Influences Excitatory Neuromuscular Response of Isolated Small Intestine Preparations

**Figure 3:**
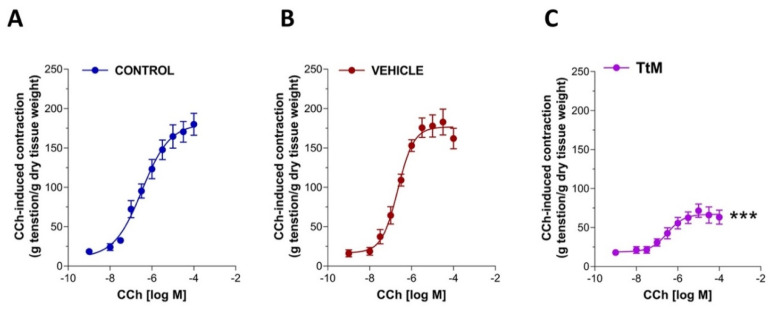
*Tilia tomentosa* Moench (*TtM)* extract influences ileal excitatory cholinergic response. Concentration-response curves to carbachol (CCh) in control (**A**), vehicle-treated (**B**), and 12 µg/mL *TtM*-treated (**C**) isolated ileal segments. Data are reported as mean ± SEM. Vehicle = Ethanol. *** *p* < 0.001 vs. control.

Considering the influence of *TtM* on the spontaneous small intestine activity, we evaluated *TtM*-mediated effects on ileal cholinergic response. Therefore, isolated ileal segments were preincubated for 15 minutes with 12 µg/mL *TtM* and then were subjected to cumulative addition of carbachol (CCh), a full cholinergic agonist. As shown in Figure 3, the pretreatment with *TtM* caused a marked reduction of CCh-induced contraction compared to the control or EtOH-treated ileal segments (Emax = −62 ± 3.5%; *p* < 0.001). 

**Figure 4:**
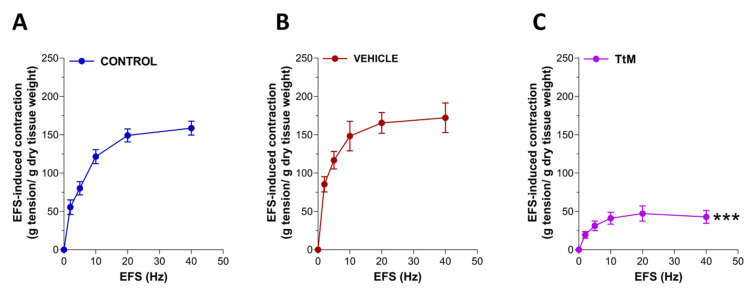
*Tilia tomentosa* Moench (*TtM)* extract influences ileal excitatory neuromuscular response. Neuromuscular excitatory response induced by Electric Field Stimulation (EFS; 0–40 Hz) in control (**A**), vehicle-treated (**B**), and 12 µg/mL *TtM*-treated (**C**) isolated ileal preparations. Data are reported as mean ± SEM. Vehicle = Ethanol. *** *p* < 0.001 vs. control.

**Figure 5:**
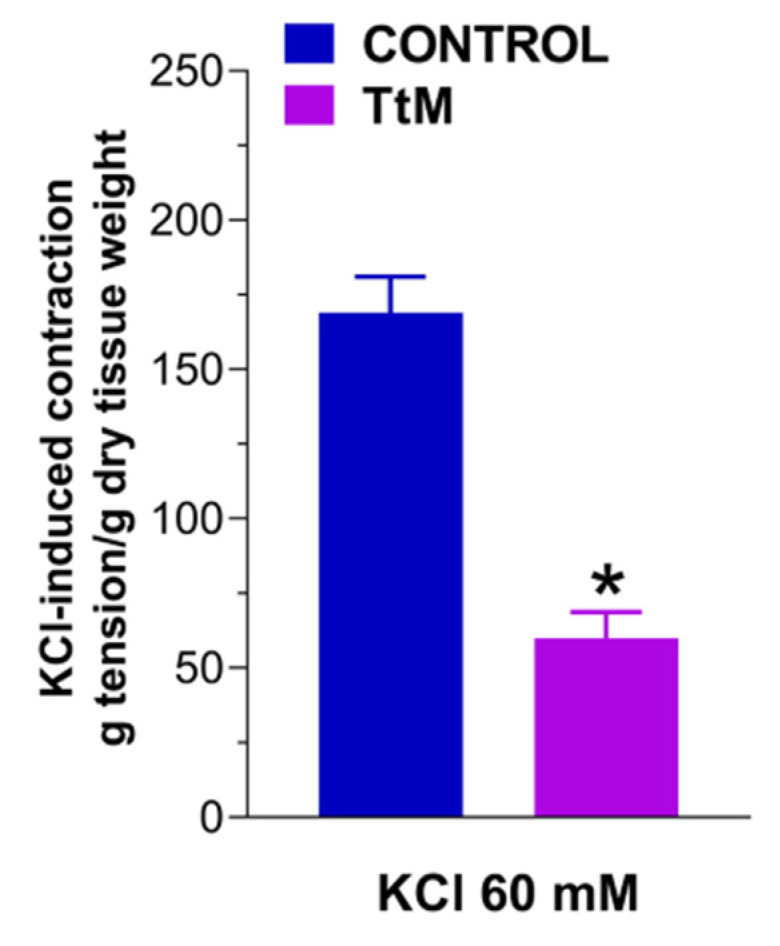
Effect of *Tilia tomentosa* Moench (*TtM)* extract on potassium chloride (KCl)-induced depolarization. Neuromuscular excitatory response induced by 60 mM KCl in control and 12 µg/mL *TtM*-treated isolated ileal preparations. Data are reported as mean ± SEM. * *p* < 0.05 vs. control.

Furthermore, preincubation with *TtM* determined a strong reduction of the neuromuscular response induced by increasing frequency of EFS compared to the control or ethanol-treated ileal segments (Emax = −72 ± 1.5%; *p* < 0.001; Figure 4). The response to high potassium-induced depolarization was affected by pretreatment with *TtM* (by 60 ± 2.5%; Figure 5).

**Figure 6:**
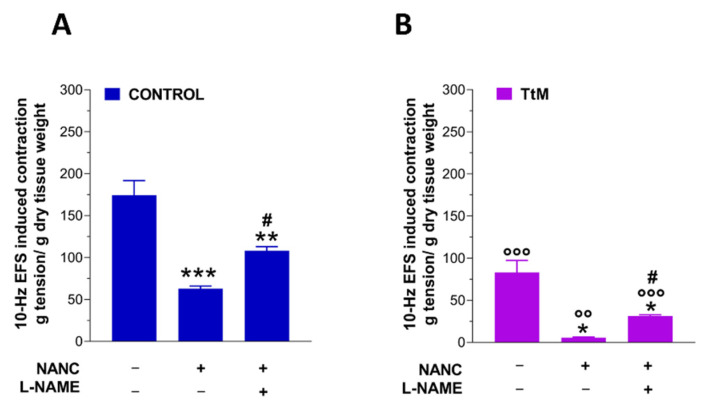
*Tilia tomentosa* Moench (*TtM)* extract influences neurally evoked tachykininergic contractions. (**A–B**) Neuromuscular excitatory response induced by 10 Hz EFS in absence or presence of non-adrenergic non-cholinergic (NANC) conditions or Nω-Nitro-L-arginine methyl ester hydrochloride (L-NAME) in control (**A**), and 12 µg/mL *TtM*-treated (**B**) isolated ileal preparations. Data are reported as mean ± SEM. The symbols “ **−”** or “+” stand for “in absence” or “in presence” of NANC and/or L-NAME, respectively. * *p* < 0.05, ** *p* < 0.01, *** *p* < 0.001 vs. respective control; °° *p* < 0.01, °°° *p* < 0.001 vs. control; # *p* < 0.05 vs. respective control in NANC conditions.

To better understand the impact of *TtM* on other enteric excitatory pathways besides the cholinergic one, we evaluated the effect of *TtM* on tachykininergic neurotransmission. To uncover the tachykininergic response, isolated ileal segments were exposed to NANC conditions through the addition of atropine, a muscarinic receptor antagonist, and guanethidine, an adrenergic blocker, in the presence or absence of L-NAME, a pan-NOS inhibitor, and exposed to 10 Hz EFS. As shown in Figure 6, pretreatment with *TtM* caused a significant reduction of 10 Hz-induced contraction that was almost abolished in the presence of NANC conditions and was partially recovered by L-NAME pretreatment. 

### 3.4. Tilia Tomentosa Induces Inhibitory Neuromuscular Responses of Isolated Small Intestine Preparations

**Figure 7:**
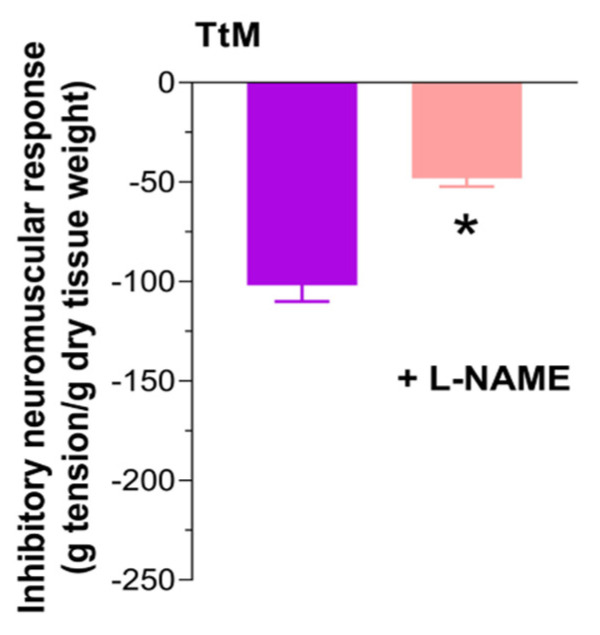
*Tilia tomentosa* Moench (*TtM)* extract influences ileal inhibitory response. 12 µg/mL *TtM*-mediated relaxation in absence or presence of Nω-Nitro-L-arginine methyl ester hydrochloride (L-NAME) in isolated ileal preparations. Data are reported as mean ± SEM. The symbol “+” stands for “in presence” of L-NAME. * *p* < 0.05 vs. respective control.

Since changes in the excitatory response are generally dependent on a higher contribution of inhibitory neurotransmission, such as that mediated by nitrergic pathways, we assessed the *TtM*-mediated relaxation in the absence or presence of L-NAME. Pretreatment with L-NAME caused a marked reduction of the relaxation induced by *TtM*, suggesting an involvement of nitric oxide (NO) in the relaxation mediated by *TtM* (−50%; *p* < 0.05; Figure 7).

**Figure 8:**
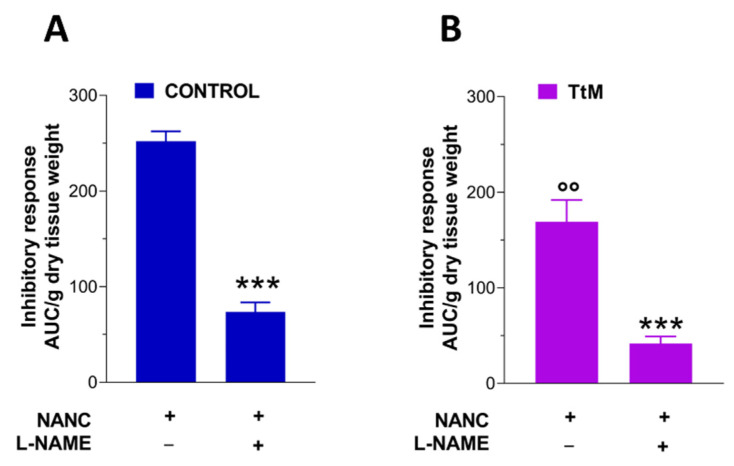
*Tilia tomentosa* Moench (*TtM)* extract influences nitric oxide-mediated relaxation. (**A**) 10 Hz EFS-evoked relaxation in non-adrenergic non-cholinergic (NANC) conditions with or without Nω-Nitro-L-arginine methyl ester hydrochloride (L-NAME) in control (**A**) and 12 µg/mL *TtM*-treated (**B**) isolated ileal segments. Data are reported as mean ± SEM. The symbols “**−”** or “+” stand for “in absence” or “in presence” of NANC and/or L-NAME, respectively. *** *p* < 0.001 vs. respective control; °° *p* < 0.001 vs. control.

To better define the contribution of the nitrergic neurotransmission, we tested the impact of *TtM* on NO-mediated relaxation induced by 10 Hz EFS stimulation in NANC conditions. As shown in Figure 8, pretreatment with *TtM* extract markedly reduced 10 Hz EFS-induced relaxation of isolated ileal segments in NANC conditions compared to the control. Pretreatment with the pan-NOS inhibitor L-NAME almost completely blocked EFS-evoked NANC relaxation in *TtM*-treated preparations. Conversely, in control ileal segments, this response was only partially abolished by L-NAME (Figure 8).

### 3.5. Tilia Tomentosa Does Not Affect Myenteric Plexus Structure of Isolated Small Intestine Preparations

**Figure 9:**
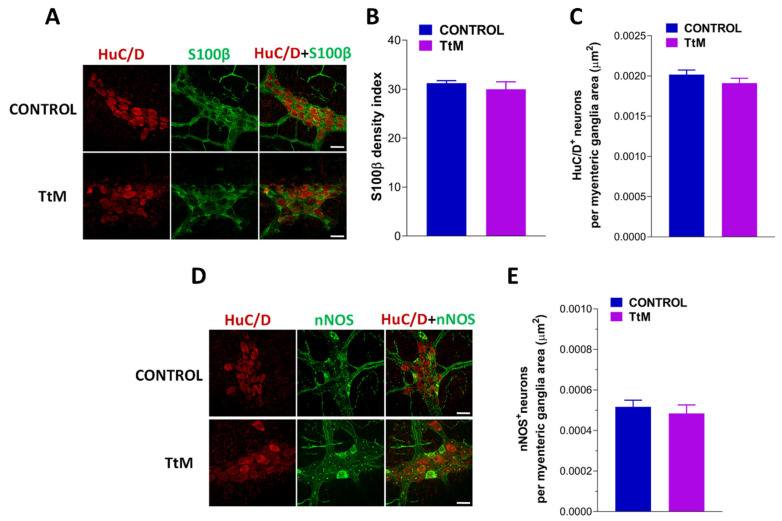
*Tilia tomentosa* Moench (*TtM)* extract maintains enteric nervous system morphology. (**A**, **D**) Representative confocal microphotographs showing the distribution of HuC/D^+^ neurons (**A** and **D**; red), S100β^+^ glial cells (**A**; green) and nNOS^+^ neurons (**D**; green) in longitudinal muscle-myenteric plexus (LMMP) preparations in presence of 12 µg/mL *TtM* pretreatment (bars = 22 µm). (**B**) Changes in S100β density index in LMMP preparations in presence of 12 µg/mL *TtM* pretreatment. (**C**–**E**) Analysis of HuC/D^+^ (**C**) and nNOS^+^ (**E**) neurons in ileal LMMPs in presence of *TtM* pretreatment. Data are reported as mean ± SEM.

Considering the changes in the enteric neurotransmission, we further evaluated the influence of *TtM* in vitro treatment on the myenteric neuroglial network, to exclude any potential acute damage on the ENS [15,16]. No changes in the immunofluorescence distribution of the glial marker S100β as well as on the number of HuC/D^+^ and nNOS^+^ neurons were found in ileal preparations treated with the extract compared to the control (Figure 9).

## 4. Discussion

GI functions are sustained by several neurotransmitters released by the ENS, such as acetylcholine and tachykinins, which mediate the excitatory contraction, or by Nom which is involved in the inhibitory relaxation. The combination of contraction and relaxation determines gut motility [24,25]. In presence of FGDIs, gut function is altered, and patients report a variety of symptoms, including motility disturbance, visceral hypersensitivity, altered mucosal and immune function, as well as changes in the gut microbiota [26,27,28]. The pathophysiology of FGIDs is still not fully understood, and various complex mechanisms appear to be implicated, mostly determining dysmotility [29,30]. In this regard, several plant formulations have been proposed as treatment options to improve the FGIDs by acting as a spasmolytic, carminative, or analgesic [5,28,31]. Accordingly, the effects of plant derivatives on GI activity should be thoroughly evaluated as a necessary step for determining the benefits of *TtM* use in FGIDs. Considering current legal directives and civil society’s opinions, investigations based on alternative models are favored over classical animal studies. In accordance with the 3R principles for animal welfare, substantial reduction in animal use was effectively achieved by performing coordination (or coordinated reduction) [32]. In the case of gut motility, in vivo assays have been successfully substituted by in vitro/ex vivo techniques that rely on isolated intestinal segments, which offer the opportunity to assess spontaneous and receptor/non-receptor-mediated contractility when preserved under conditions replicating those in vivo [33,34]. 

Since motor disturbances of the small bowel are increasingly involved in the pathogenesis of FGIDs, isolated ileal preparations were used as an ex vivo model to investigate the pharmacological effects of *TtM* on intestinal motor function and to characterize its mechanism of action [35]. HPLC analysis of *TtM* extract resulted in the identification of quercetin and kaempferol as dominant aglycones, as previously described [36]. In addition to the direct antioxidant activity, these compounds were shown to exert beneficial effects in the GI tract. Quercetin and kaempferol have been revealed to ameliorate GI function in the presence of IBS for their ability to inhibit lipopolysaccharide and NF-kB signaling [37,38], respectively. Moreover, administration of quercetin and kaempferol was shown to ameliorate gut the microbiota composition affected by colitis or high fat diet-induced obesity, to highlight the wide range of beneficial biological actions mediated by these flavonoids [39,40].

Dietary flavonoids (e.g., kaempferol and quercetin) as well as their aromatic metabolites appear to modify the gut microbial community by displaying prebiotic effects and antimicrobial activity. These properties are of paramount importance in inflammatory bowel disease, since dietary flavonoids can modulate gut microbial diversity towards a beneficial species-rich gut ecosystem [40,41].

We show here, for the first time, that *TtM* has the following consequences on GI contractility: (i) an enhanced motor inhibitory effect sensitive to L-NAME; (ii) a marked reduction of excitatory neuromuscular response, mainly affecting cholinergic and tachykininergic neurotransmission; (iii) a reduced NANC inhibitory relaxation, with no changes in the phenotype of the neuroglia network.

We first evaluated the influence of *TtM* on ileal basal contractility, obtaining a marked inhibition of the ileal basal tone and revealing the relaxant properties of the plant extract. Several pharmacological mechanisms are involved in limiting excessive intestinal smooth muscle contractility, including antagonism of cholinergic receptors or tachykininergic receptors and stimulation of NO production or β-adrenergic pathways. Interestingly, *TtM* exerted an inhibitory effect on cholinergically mediated contractions of the mouse small intestine evoked by EFS, which induces the release of neuronal acetylcholine, or by the non-selective cholinergic agonist carbachol, which interacts primarily with cholinergic receptors, expressed mainly on smooth muscle cells. 

The application of *TtM* also relaxed the smooth muscle of mouse ileum when contracted in a sustained manner following the addition of KCl to the bathing solution. Such a technique is usually considered to cause contraction of GI preparations, largely by depolarization of the smooth muscle, with the consequent opening of L-type calcium channels and a rise in intracellular calcium, leading to smooth muscle contraction [17,22,42,43]. Thus, the ability of *TtM* to inhibit KCl-induced contraction suggests a direct action on the smooth muscle cells. In this response, the potency of *TtM* appeared to be reduced compared to that measured when tested against the EFS-evoked contractions. However, it is difficult to know with any certainty if such differences are due to the differences in types of assays (e.g., analysis of phasic contraction versus tonic contraction), or if these responses imply an additional ability of *TtM* to directly inhibit neuronal functions. Indeed, we found that up to 10 Hz, acetylcholine is the main neurotransmitter involved in ileal contraction, whereas, increasing the frequency, other excitatory neurotransmitters are engaged, such as tachykinins [17]. The enteric tachykininergic system has a key role in the maintenance of the intestinal neuromuscular function, and alterations in tachykinin-mediated neurotransmission may participate in the pathogenesis of FGIDs [19,23,44]. Intriguingly, *TtM* reduced the enteric tachykinin-mediated responses in isolated ileal segments. These findings highlight the potential effect of *TtM* on excitatory neurotransmission, resulting in a spasmolytic effect. Therefore, based on the evident relaxant properties, we further evaluated the role of the nitrergic neurotransmission on the *TtM*-induced relaxation. Indeed, in the presence of L-NAME, a pan-NOS inhibitor, *TtM* per se showed a reduced inhibitory effect of the ileal basal contractility, confirming the involvement of NO in the *TtM*-induced relaxation. A similar result was obtained by Gharzouli and Holzer when testing several flavonoids on guinea pig small intestine contractility; they found that the antiperistalsis effect of quercetin was partially prevented by pretreatment with L-NAME [45]. 

Overall, based on the present findings, it is plausible to hypothesize that *TtM*, antagonizing cholinergic and tachykininergic neurotransmission and enhancing NO-mediated response, could contribute to the modulation of the small intestinal motor abnormalities occurring in presence of several diseases, including FGDIs [46,47]. In this regard, considering that plants belonging to the *Tilia* genus produce compounds, such as flavonoids, that have been shown to exert anti-neuroinflammatory and antiproliferative activities in an in vitro model [48], we further focused our attention on testing the effect of *TtM* on the small intestine myenteric neuroglial network by whole-mount immunohistochemistry, which has a crucial role in the development of microinflammatory processes predisposing to neuroplastic adaptive changes [15]. However, no changes in the glial marker S100β and on the neuronal phenotype were observed to confirm the potential protective role of *TtM* on ENS.

## 5. Conclusions

These findings provide a pharmacological basis for the traditional use of *TtM* in FGIDs, such as IBS. *TtM* contains many phenolic and flavonoid compounds, including quercetin, kaempferol, tiliroside, astragalin, and isoquercitrin [49]. It has been reported that quercetin reduces peristalsis [45], whereas kaempferol impairs intestinal transit in mice through α-2 adrenergic receptors and calcium channels [50]. However, flavonoids appear also to be involved in NO production and in the inhibition of calcium and potassium channels, leading to endothelium smooth muscle relaxation [51].

In conclusion, our findings advocate for a *TtM*-induced relaxation effect on isolated ileum in mice, which involves NO and tachykininergic pathways. This spasmolytic response is possibly due to quercetin, kaempferol, and/or other flavonoid content present in the *TtM* extract, highlighting the potential role of *TtM* in FGIDs.

## Figures and Tables

**Table 1:** Primary and secondary antibodies and their respective dilutions used for immunohistochemistry on ileal longitudinal muscle-myenteric plexus whole-mount (LMMP) preparations.

Antibody	Host Species	Dilution	Catalog Number	Source
*Primary Antisera (Clone)*				
HuC/D (16A11)	Mouse biotin-conjugated	1:100	A-21272	Thermo Fisher Scientific (Monza, Italy)
nNOS (polyclonal)	Rabbit	1:100	61-700	Thermo Fisher Scientific
S100β (P50114)	Guinea pig	1:100	287 00	Synaptic Systems, (Göttingen, Germany)
*Secondary Antisera*				
Goat anti-rabbit IgG Alexa 488-conjugated	-	1:1000	A-11008	Thermo Fisher Scientific
Goat anti-guinea pig IgG Alexa Fluor 488-conjugated	-	1:1000	AB_2534117	Thermo Fisher Scientific
Streptavidin Alexa 555-conjugated	-	1:1000	S21381	Thermo Fisher Scientific

## Data Availability

The data presented in this study are available on request from the corresponding author.
